# Advancements in alternative approaches to address antimicrobial resistance in bacterial pneumonia: a comprehensive review

**DOI:** 10.3389/fmicb.2025.1704931

**Published:** 2025-11-19

**Authors:** Dala N. Daraghmeh, Roaa Waleed AbuIriban, Nour Nawawreh, Ansam Mahmoud Abuamro, Momin M. Alassar, Salahaldeen N. Daraghma, Nesma M. Alhajahmed, Yasmeen Thandar

**Affiliations:** 1 College of Public Health, Al-Quds University, Jerusalem, Palestine; 2 Pharmaceutical Research and Innovation Hub, Department of Pharmacology, Faculty of Pharmacy, Al-Quds University, Jerusalem, Palestine; 3 Al Awda Health and Community Association, Gaza, Palestine; 4 United Nations Relief and Works Agency, Gaza, Palestine; 5 Ministry of Health, Gaza, Palestine; 6 College of Dentistry, Arab-American University, Jenin, Palestine; 7 Department of Basic Medical Sciences, Durban University of Technology, Durban, South Africa

**Keywords:** AMR, MDR, bacterial pneumonia, phage, probiotics, stem cells

## Abstract

**Purpose:**

This review explores both current and emerging alternative treatment approaches to combat AMR specifically in the context of bacterial pneumonia, highlighting therapies that extend beyond conventional antibiotics.

**Methods:**

PubMed, Embase, and Google Scholar were searched for full-text, English-language articles, with emphasis on publications from 2020 to 2025. Earlier seminal studies were also included when necessary to provide historical, mechanistic, or conceptual context. The review focuses was on alternative strategies that have shown effectiveness in preclinical or clinical settings to combat AMR in relation to bacterial pneumonia.

**Results:**

Emerging strategies to tackle AMR in bacterial pneumonia involve several innovative approaches including stem cells, bacteriophage therapy, metal based nanoparticles (e.g., silver, copper, and gold). The adjunctive use of probiotics and herbal medicine has demonstrated potential in enhancing clinical outcomes and modulating host immunity. Moreover, gene editing technologies like CRISPR-CAS and various vaccination programs are being investigated for their roles in prevention and resistance management. While these methods show promise, many are still in the early stages of development and encounter challenges related to standardization, safety, and regulatory approval.

**Conclusion:**

Alternative therapies present exciting possibilities for addressing AMR in bacterial pneumonia. However, to effectively translate these innovations into clinical practice, we need thorough research, international collaboration, and supportive policy frameworks. By combining these strategies with antimicrobial stewardship initiatives, we can help maintain antibiotic effectiveness and enhance patient outcomes.

## Introduction

The ongoing rise of antimicrobial resistance (AMR) is a critical global health threat, driven by overuse in healthcare, agriculture, and the environment (Urban-Chmiel et al., 2022). This has led to the growth of multidrug-resistant organisms (MDRs), threatening treatment efficacy for bacterial infections (Abbas et al., 2024; Salam et al., 2023). The World Health Organization (WHO) reported that AMR was associated with 1.27 million deaths globally in 2019, with projections to reach 10 million by 2050 without effective intervention (World Health Organization, 2024). However, a recent modeling analysis presented an even more alarming scenario, predicting that up to 92 million lives could be lost between 2025 and 2050 due to inadequate management of severe infections and limited access to antibiotics (Naghavi et al., 2024). This growing issue risks pushing us back to the preantibiotic era, exacerbating public health and economic challenges (Urban-Chmiel et al., 2022).

Bacterial pneumonia continues to be a leading cause of morbidity and mortality, particularly among vulnerable groups such as children, the elderly, and immunocompromised individuals (İlerler et al., 2025; Jansen et al., 2021; Mittapally et al., 2018). The presence of AMR in bacterial pneumonia infection complicates treatment protocols, prolongs hospital stays, increases healthcare costs, and elevates mortality rates (İlerler et al., 2025).

While global efforts continue to promote rationale antibiotic use, infection prevention and control, vaccination, and new antibiotic development, there is an urgent need to explore alternative therapeutic approaches that can bypass conventional resistance mechanisms (Jansen et al., 2021; Mittapally et al., 2018).

In this review, we explored recent advancements in alternative strategies that can effectively combat bacterial pneumonia while circumventing the growing challenge of AMR. By evaluating recent research and developments, this review seeks to highlight promising alternatives such as bacteriophage therapy, mesenchymal stem cells (MSCs), metal nanoparticles, probiotics, CRISPR-Cas systems, vaccines, and natural remedies. This comprehensive review aims to provide insights into potential breakthroughs that could reshape the future of bacterial pneumonia treatment, ultimately improving patient outcomes and preserving the efficacy of existing antibiotics.

## Methodology

PubMed, Embase, and Google Scholar were searched for English-language full-text articles using the following terms: pneumonia, antimicrobial, resistance, MDR, alternatives, metals, bacteriophages, natural remedies and stem cells, natural, herbal, plant, botanical, phytotherapy.

The search primarily focused on publications from 2020 to 2025 to capture recent advancements, while earlier seminal studies were also included when relevant to provide historical, mechanistic, or conceptual context related to antimicrobial resistance and bacterial pneumonia.

Articles were included if they were published in English, focused on antimicrobial resistance or alternative therapeutic strategies in bacterial pneumonia, and presented relevant experimental, clinical, or mechanistic evidence. Studies were excluded if they were non-English, unrelated to bacterial pneumonia or AMR, or were conference abstracts, commentaries, or editorials without full-text data.

### Pneumonia pathogenesis

**Figure 1:**
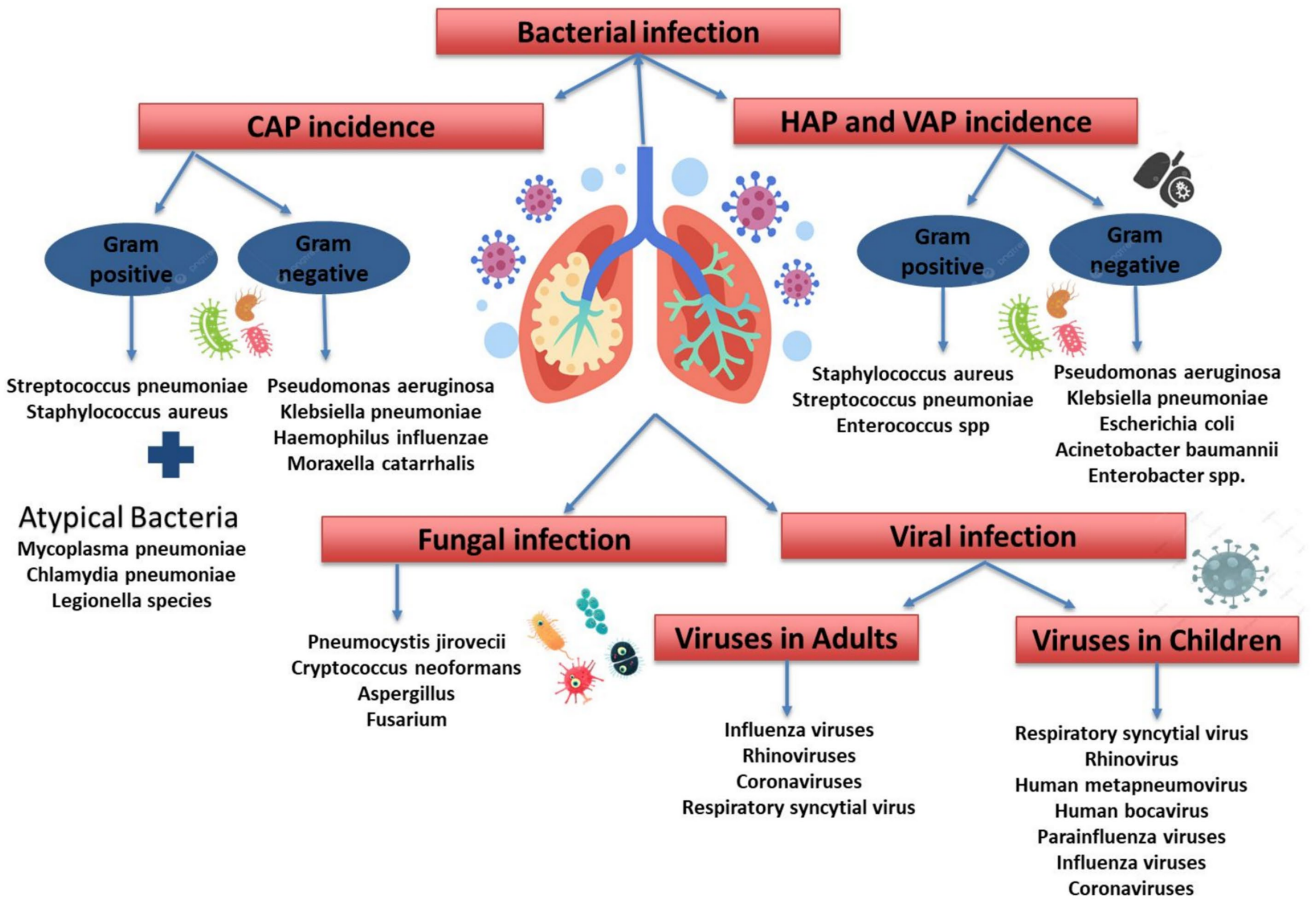
Etiological classification of pneumonia. CAP, community acquired pneumonia; HAP, hospital acquired pneumonia; VAP, ventilator-associated pneumonia.

Pneumonia is a common acute respiratory infection affecting the lungs, causing inflammation and fluid accumulation in the alveoli. It is a leading cause of mortality worldwide, especially in children under 5 and the elderly (Sabbagh et al., 2024; Torres et al., 2021). Different clinical forms of pneumonia—community-acquired (CAP), hospital-acquired (HAP), and ventilator-associated (VAP)—exhibit distinct antimicrobial resistance patterns, underscoring the need for innovative therapeutic strategies beyond traditional antibiotics (Figure 1; Torres et al., 2021).

Understanding pneumonia classifications aids in diagnosis and treatment, as it can be caused by bacteria, viruses, and fungi (Figure 1). Since the prevalence of these causes varies by region, this review focuses on bacterial pneumonia. Common bacterial causes for CAP includes Gram-Positive Bacteria [i.e., *Streptococcus pneumoniae (S. pneumoniae), Staphylococcus aureus (S. aureus)*], Gram-Negative Bacteria [*Pseudomonas aeruginosa (P. aeruginosa), Klebsiella pneumoniae (K. pneumoniae), Haemophilus influenzae (H. influenzae), Moraxella catarrhalis*], and Atypical Bacteria [i.e., *Mycoplasma pneumoniae (M. pneumoniae), Chlamydia pneumoniae (C. pneumoniae), Legionella species*]. Furthermore, the bacterial cause for HAP and VAP includes Gram-Positive Bacteria (*S. aureus, S. pneumoniae, Enterococcus* spp.). Gram-Negative Bacteria [*P. aeruginosa, K. pneumoniae, Escherichia coli (E. coli), Acinetobacter baumannii, Enterobacter* spp.] (Jain et al., 2023).

Pneumonia involves pathogen invasion and an inflammatory response in the alveoli (Korkmaz and Traber, 2023), involving key cells likr alveolar macrophages, neutrophils, and pulmonary epithelial cells. Alveolar macrophages trigger inflammation by releasing pro-inflammatory cytokines like tumor necrosis factor-alpha (TNF-α), IL-1β, and IL-8 to recruit neutrophils, which can also cause tissue damage through the release of toxins. The severity of pneumonia varies by pathogen and inflammation balance, with differences observed between Gram-positive and Gram-negative bacteria (Torres et al., 2021; Korkmaz and Traber, 2023). Understanding these immune mechanisms is crucial for developing targeted pneumonia therapies.

### Antibiotic history and resistance evolution

**Figure 2:**
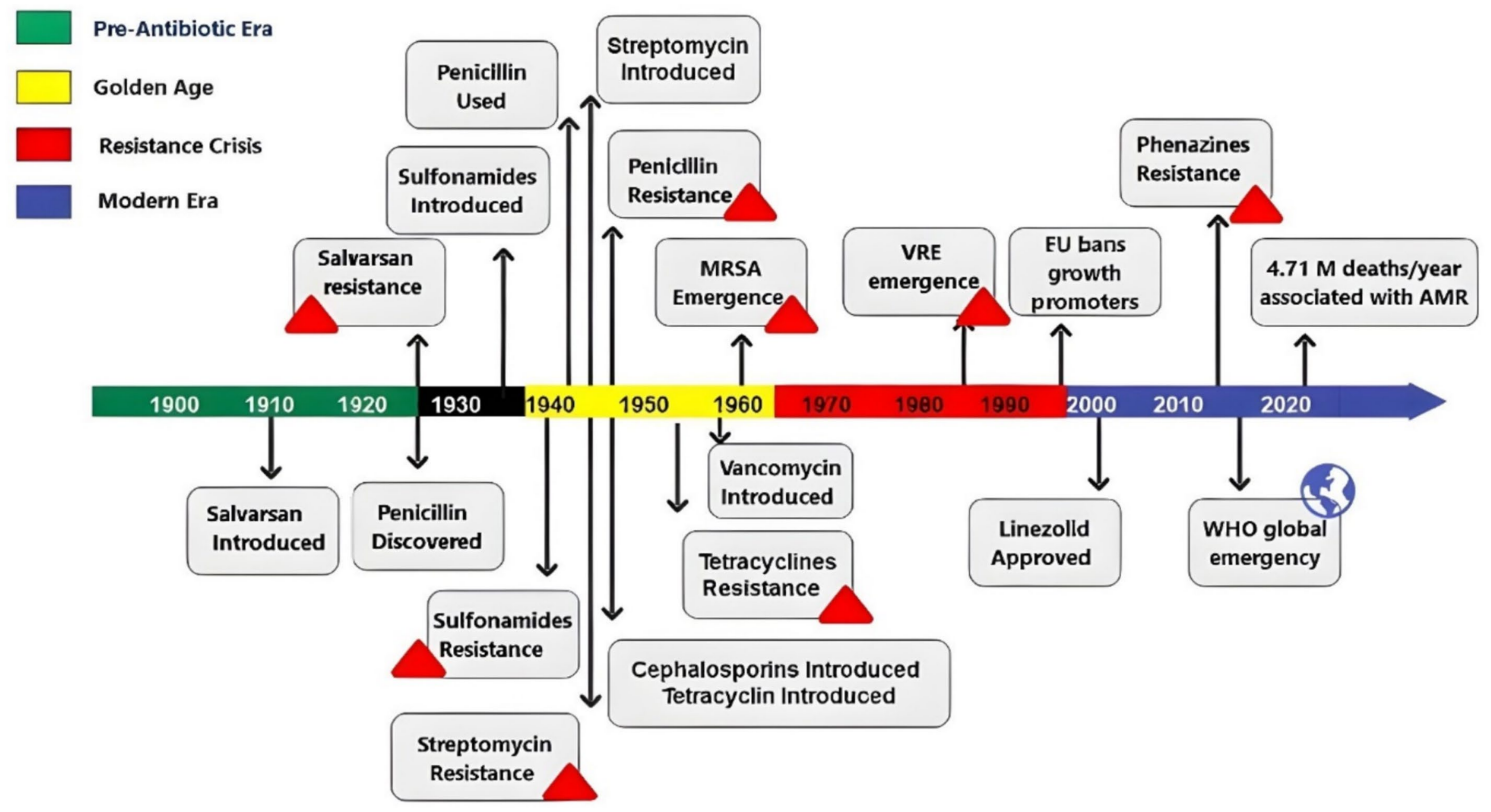
Timeline showing the antibiotic history and resistance evolution through the last century. MRSA, methicillin-resistant *Staphylococcus aureus*; VRE, vancomycin-resistant enterococci; EU, European Union; WHO, World Health Organization.

The discovery of antibiotics revolutionized medicine, began with Alexander Fleming’s observation of penicillin in 1928. The true launch of the antibiotic era occurred in the 1940s, ushering in a golden age of drug discovery marked by the rapid development of most current antibiotic classes (Christensen, 2021). However, widespread use of antibiotics quickly resulted in the emergence of resistance—with penicillin resistance appeared just 4 years after its clinical introduction. Resistance to other antibiotics followed: tetracycline (introduced in 1945) saw resistance by 1953, and vancomycin (introduced in 1958) was followed by vancomycin-resistant enterococci (VRE) by 1988 (Hutchings et al., 2019; Stennett et al., 2022; Hunt and Kates, 2024). The rise of MD bacteria, including methicillin-resistant *Staphylococcus aureus* (MRSA), first identified in 1961, further deepened the crisis. Compounding the problem, antibiotics were also used extensively in agriculture—as growth promoters in livestock feed. This practice, which created selective pressure for resistant strains, prompted the European Union to ban antibiotic growth promoters in 1999 (Casewell et al., 2003). In 2021, this growing issue was linked to an estimated 4.71 million deaths globally due to bacterial AMR, with 1.14 million of these deaths being directly attributable to bacterial AMR (Naghavi et al., 2024; World Health Organization, 2024; World Health Organization, 2015). A timeline of antibiotic history and resistance is illustrated in Figure 2 (Hutchings et al., 2019; Stennett et al., 2022; Hunt and Kates, 2024; Naghavi et al., 2024).

### Conventional and novel antibiotics

Recent research highlights the growing challenge of MDR bacteria in treating pneumonia, complicating options (Torres et al., 2021). Traditional antibiotics like beta-lactams, macrolides, quinolones, carbapenems, and aminoglycosides, face resistance for many strains. Supplementary Table 1 summarizes essential antibiotic classes used in treating CAP and HAP, outlining their subclasses, uses, resistance mechanisms, and associated genes (Russo, 2020; Baran et al., 2023; Lee et al., 2018; American Thoracic Society; Infectious Diseases Society of America, 2005).

The rise of AMR necessitates the development of targeted therapies informed by resistance profiles, yet empiric therapy is still common due to unidentified infection sources. This highlights the urgent need for innovative broad-spectrum antibiotics that specifically target resistant strains (American Thoracic Society; Infectious Diseases Society of America, 2005).

Recent advancements have introduced promising antibiotics for treating bacterial pneumonia. Notably, Ceftobiprole and Ceftaroline, both broad-spectrum cephalosporins, have recently been approved for treating HAP and CAP (U.S. Food and Drug Administration, 2024). Additionally, Ceftolozane combined with tazobactam, a new beta-lactam/beta-lactamase inhibitor, has demonstrated potent activity against MDR and extensively drug-resistant (XDR) bacteria, such as *P. aeruginosa*. Solithromycin, a novel fluoroketolide, which belongs to a next-generation subclass of macrolides showed its effectiveness in treating macrolide-resistant CAP pathogens, including *S. pneumoniae, H. influenzae*, and atypical pathogens, making it a promising first-line treatment for CAP due to its established safety and efficacy (Russo, 2020).

Additionally, the FDA has approved Omadacycline, an aminomethylcycline, and Lefamulin, from the pleuromutilin class, for treating CAP. A recent systematic review and meta-analysis concluded that both antibiotics exhibit comparable strong activity against gram-positive strains (Wu et al., 2024).

Current research into novel antibiotics highlights advantages such as broad-spectrum activity, reduced toxicity, and improved lung tissue penetration. Combinations like Ceftaroline/avibactam and Ciprofloxacin/Murepavadin have shown promise in preliminary studies (Jia et al., 2022; Wei et al., 2024).

Ongoing clinical trials and global initiatives emphasize collaboration among stakeholders in antibiotic research. Advancements in diagnostics and public education are vital for combating AMR and ensuring the effectiveness of antibiotics in the future.

### Artificial intelligence in AMR prediction and drug discovery

Artificial intelligence (AI) is playing a transformative role in conbating AMR by enhancing existing therapeutic stratigies and improving resistance managment. AI models, especially deep learning and machine learning, can accelerate the drug discovery pipeline through mining extensive chemical and genomic databases to identify promising antibacterial drugs and predict molecular interaction, pharmacokinetics properties, and safety profiles during early development stages (Mulat et al., 2025).

In 2020, Stokes et al. from Massachusetts Institute of Technology’s Collins Lab used a deep learning approach to screen different molecular structures from the Drug Repurposing Hub, leading to the discovery of Halicin, a broad-spectrum antibiotic that demonstrated efficacy in mice against *pan-resistant Acinetobacter baumannii* (Stokes et al., 2020).

Moreover, AI assists in resistance gene prediction by processing and analyzing genomic data to detect mutations associated with drug resistance. While this analysis was previously a complex, machine learning and deep learning now outperform conventional methods, providing more comprehensive predictive models (Li et al., 2024). For example, Condorelli et al. used six machine learning models to predict AMR in *K. pneumoniae* using Biometec and public datasets containing different strains and resistance genes. Gradient boosting and k-nearest neighbor classifiers achieved the highest accuracy, with the Biometec dataset showing an accuracy of 83%, and the public dataset achieved over 90% for nearly all models (Condorelli et al., 2024).

AI also has proven highly effective in the design and optimization of antimicrobial peptides (AMPs), short amino acid sequences that act as antibiotic alternatives. Since many natural AMPs are toxic to mammalian cells, AI driven models are increasingly used to design novel AMPs with enhanced potency and reduced cytotoxicity. Le et al. applied a peptide language-based deep generative model to design broad-spectrum AMP candidates, with over 90% demonstrating inhibitory activity against bacteria such as *S. aureus, K. pneumoniae*, and *P. aeruginosa*. Notably, these designed AMPs did not induce resistance in *S. aureus* over time, unlike ciprofloxacin (Li et al., 2024).

Collectively, these advances underscores how AI driven approaches in drug discovery, resistance prediction, design novel AMPs, provide an impactful stratigies to address and control resistance in bacterial pneumonia.

### Plasmids in antimicrobial resistance and gene editing

Plasmids are extrachromosomal DNA that facilitate the spread of antimicrobial resistance genes (ARGs) among bacteria through horizontal gene transfer (Rozwandowicz et al., 2018). This is particularly evident in *K. pneumoniae*, a major cause of healthcare-associated infections. *Incompatibility group F (IncF)* plasmids within *K. pneumoniae* often carry the blaCTX-M-15 gene, which confers resistance to third-generation cephalosporins (Ikhimiukor et al., 2024). Dissemination of ARGs occurs through conjugation, transposition, and recombination (Table 1; Shen et al., 2022).

**Table 1:** Plasmid-driven resistance mechanisms and strategic countermeasures.

Mechanism of resistance	Description	Strategy to combat
Conjugation	Direct transfer of plasmids between bacteria via type IV secretion systems and pili.	Use conjugation inhibitors CRISPR-Cas to target plasmid transfer.
Transposition	Integration of mobile genetic elements (e.g., transposons) into plasmids, facilitating ARG spread.	Inhibit transposase activity and employ gene-editing strategies to disrupt transposons.
Recombination	Acquisition and rearrangement of gene clusters through homologous recombination.	Surveillance with WGS implement regulatory measures like antibiotic stewardship

ARG, antimicrobial resistance genes; WGS, whole-genome sequencing.

Conjugation through Type IV secretion systems on plasmids like IncF, enables horizontal ARG transfer among isolates of *K. pneumoniae*. Transposition, Mobile genetic elements like transposons (e.g., Tn3 carrying blaTEM-1) integrate ARGs into plasmids, while homologous recombination can produce hybrid plasmids (e.g., IncHI-IncN in *K. pneumoniae*) that expand resistance profiles (Table 1; Shen et al., 2022; Boueroy et al., 2022).

Plasmid-borne resistance genes such as blaNDM-1, blaKPC, and mcr-1 are linked to treatment failures in patients with bacterial pneumonia. These genes render standard therapies, including carbapenems and colistin, ineffective (Boueroy et al., 2022). The presence of these plasmids in high-risk clones of *K. pneumoniae* complicates outbreak control and elevates mortality risk, particularly in critical care environments (Akintayo et al., 2025).

Several strategies have been proposed to address AMR by targeting plasmids and other mobile genetic elements through various methods, including CRISPR-Cas systems, conjugation inhibitors, and enhancing surveillance via whole-genome sequencing (WGS). CRISPR-Cas system, this is a gene editing tool that can specifically target and cleave DNA sequences that encode antibiotic resistance genes, demonstrating potential for controlling horizontal gene transfer and limiting the spread of antibiotic resistance (Fursova et al., 2022). Conjugation Inhibitors, Synthetic peptides are designed to disrupt plasmid transfer mechanisms and have shown promise in preventing the dissemination of resistance genes during bacterial infections (Svara and Rankin, 2011). Enhanced Surveillance, WGS and plasmid typing improve the ability to track the spread of resistance, aiding in timely interventions and informing antibiotic stewardship programs (Ajayi et al., 2024).

Plasmids play a crucial role in the propagation of AMR in bacterial pneumonia. Understanding their mechanisms of action and clinical impacts is essential for developing alternative treatment and containment strategies. By integrating novel technologies like CRISPR, along with rigorous surveillance and policy reforms, we can offer a promising path toward reducing AMR and improving patient outcomes in pneumonia treatment (Table 1).

### Alternatives to antimicrobials

With the global rise of AMR threatening to render existing antibiotics ineffective, the urgent pursuit of safe and sustainable alternatives is no longer optional -it is essential for the future of infectious disease management. This has led to increased research on MSCs, bacteriophages, metal nanoparticles, probiotics, herbal medicine, vaccines, and gene editing technology. To ensure clarity and logical progression, this review is organized according to the nature and mechanism of each therapeutic approach, beginning with cellular and biological therapies, followed by material and molecular-based strategies, and concluding with naturally derived and complementary options.

Table 2 offers a comparative overview of these alternative therapies, detailing their mechanisms, advantages, safety profiles, and research stages, with additional details in the following sections.

**Table 2:** Therapeutic strategies against antimicrobial resistance: mechanisms, benefits, and development stages.

Therapy	Mechanism of action	Advantages	Safety	Current stage
CRISPR-Cas Systems	Targets resistance genes via gene editing	Precision targeting of resistance genes	Safety still under investigation	Laboratory-stage; proof-of-concept
Stem Cell- Therapies	Immunomodulation; enhances bacterial clearance	Dual anti-inflammatory and antimicrobial effects	Early investigations suggest acceptable safety profile	Animal models; early-phase clinical interest
Bacteriophage Therapy	Direct bacterial lysis Modulation of host immune responses	High specificity Low resistance risk Long-term effectiveness Ability to eliminate biofilms	Safe and tolerable in animals and humans, but there is a critical need for standardized safety assessments	Early clinical trials
Metal-Based Nanoparticles	Generates ROS; disrupts membranes; synergistic with antibiotics	Potent against MDR strains; can be combined with antibiotics	High toxicity profile	Preclinical studies; formulation development
Probiotics	Modulates gut-lung axis; inhibits pathogen colonization	Low cost; enhances host defenses	Generally safe	Mostly preclinical; some human trials
Herbal Medicine	Antimicrobial and anti-inflammatory effects	Accessible; used in traditional medicine	Some with proven safety profile but many with low evisanse	Widely used traditionally; limited clinical validation
Vaccination	Stimulates adaptive immunity Prevents infection	Prevention Reduces antibiotic use	Good safety profile	Established for some pathogens; novel vaccines in development

### Mesenchymal stem cells

Human MSCs are multipotent and self-renewing, showing significant potential in regenerative medicine and infection control. They exert strong antimicrobial effects through both direct and indirect mechanisms, particularly in the context of bacterial pneumonia. The direct antimicrobial action is largely attributed to their secretion of AMPs, such as LL-37, β-defensins, and RegIIIγ, which inhibit a wide spectrum of pathogens and help prevent biofilm formation (Byrnes et al., 2023; Park et al., 2019). A study by Qian et al. demonstrated that adipose-derived MSCs significantly reduced bacterial burden via RegIIIγ secretion in a murine *S. aureus* pneumonia model (Qian et al., 2016). These peptides, which exist in both prokaryotic and eukaryotic organisms, also offer immunogenic, anti-inflammatory, and potential anti-cancer properties.

In terms of immune regulation, MSCs primarily exert a dual immunomodulatory role: they suppress excessive inflammation while enhancing host antibacterial defenses. MSCs modulate immune responses through factors like prostaglandin E2 (PGE2), indoleamine 2,3-dioxygenase (IDO), and TNF-α, enhancing both innate and adaptive immunity. For example, lung-derived MSCs were shown to enhance phagocytosis and immune regulation in *K. pneumoniae* pneumonia models (Rangasamy et al., 2021). Furthermore, preactivated MSCs combined with antibiotics improved survival and decreased bacterial burden in rodent models of MDR *K. pneumoniae* (Byrnes et al., 2023).

MSC-derived extracellular vesicles (EVs) are gaining attention as cell-free therapy alternatives, carrying cytokines, AMPs, and miRNAs to infected tissues. In a human ex vivo lung model, MSC-EVs reduced vascular permeability and bacterial load in *E. coli* pneumonia (Park et al., 2019). Likewise, nebulized MSC-EVs improved lung histopathology and reduced inflammation in rodent pneumonia models (Gonzalez et al., 2023).

Collectively, these findings underscore MSCs and their derivatives as innovative approaches for enhancing host defenses and treating antibiotic-resistant pneumonia. However, current evidence remains primarily at the preclinical or early clinical stage, and challenges remain particularly regarding efficacy and safety as well as the delivery methods efficiency. A recent review emphasized the need for standardized protocols to mitigate risks like immune rejection, fibrosis, or long-term complications (Ahmed et al., 2024). Therefore, large scale well designed randomized controlled trials (RCTs) are essential to confirm the therapeutic safety, optimal dosing, and reproducibility of MSC-based interventions.

#### Bacteriophages

Bacteriophages, or phages, are viruses that specifically infect bacteria, discovered in 1915, predating the introduction of antibiotics (Rezaei, 2024). They consist of a protein coat enclosing nucleic acid (DNA or RNA) and are found wherever bacteria exist, with estimates of 104–108 virions per ml in water and 109 per gm in soil (Loc-Carrillo and Abedon, 2011). These viruses have a specialized structure with tunnel tails that allow them to bind to and inject their DNA into bacterial hosts (Fernandes and São-José, 2018). They play vital roles in microbial ecosystems by driving bacterial evolution and facilitating horizontal gene transfer (Drulis-Kawa et al., 2015). Their specificity and ability to replicate within and kill bacteria make them potential alternatives to antibiotics, especially amid increasing antibiotic resistance (Sahoo et al., 2024).

Phages are promising alternatives to antibiotics due to their specificity, capacity to replicate within, and ability to kill specific bacteria (Loc-Carrillo and Abedon, 2011). Unlike antibiotics, which can disrupt beneficial microbiota, bacteriophages can precisely target pathogenic bacteria while preserving microbiomes (Sahoo et al., 2024). This selective mechanism minimizes the risk of adverse side effects associated with broad-spectrum antibiotics (Loc-Carrillo and Abedon, 2011). Bacteriophage therapy uses this unique ability to treat bacterial infections resistant to traditional antibiotics (Sahoo et al., 2024). The process involves the phage attaching to specific bacterial surface receptors, injecting its genetic material, replicating within the bacterial cell, and eventually lysing the cell to release new phages that can infect additional bacterial cells (Fernandes and São-José, 2018; Drulis-Kawa et al., 2015).

Phage therapy has demonstrated significant effectiveness against key lung pathogens, including *K. pneumoniae, P. aeruginosa, Acinetobacter baumannii, S. aureus,* and *Mycobacterium abscessus*, which are linked to infections like HAP and VAP. First use in 1936, recent studies indicate that inhaled phage therapy can be effective 80–100% of the time, mainly when used as an adjunct treatment. However, challenges like phage-host specificity, manufacturing quality, and formulation stability must be addressed (Fowoyo, 2024).

Phage therapy’s effectiveness in treating respiratory infections, validated by preclinical animal models. For example, Phage 1,513, which targets *K. pneumoniae* (KP 1513), proved more effective in mice when administered at higher doses. At a high dose of 2 × 10^9^ PFU/mouse, phage 1513 attained an 80% survival rate, compared to 0% in the untreated control group. This was accompanied by reduced lung pathology, localized lesions, a decreased bacterial burden, and a significant reduction in pro-inflammatory cytokines such as TNF-α and IL-6 (*p* = 0.0159 and *p* = 0.0079, respectively) (Cao et al., 2015). Similarly, phages targeting *S. aureus* have shown greater efficacy than clindamycin in the treatment of MDR pneumonia in murine models. In VAP models, nebulized phages diminished bacterial loads and enhanced survival rates, both prophylactically and therapeutically (Fernández et al., 2021). Furthermore, phage-derived lytic enzymes, including endolysins Cpl-1, Cpl-7, and the engineered variant Cpl-711, exhibit significant bactericidal properties. These lysins significantly enhance the efficacy of antibiotics like amoxicillin or cefotaxime against *S. pneumoniae* and *S. aureus in vivo* (Fernández et al., 2021).

While clinical evidence is still emerging, it has been promising, a study involved 20 cases of phage therapy for MDR respiratory infections, revealed that 80% of patients achieved favorable outcomes, including infection resolution and heightened antibiotic susceptibility. These cases included individuals with cystic fibrosis, infections post-transplant, and VAP. Delivery methods varied, including intravenous, inhalation, and topical administration, often using multiple phages over extended periods (Khosravi et al., 2024).

Recent improvements in phage production and delivery have significantly increased the likelihood of successful phage therapy for respiratory issues. Innovative methods like spray drying and aerosolization have improved the physical stability of phage preparations, making them easier for the lungs to absorb, and facilitating inhalation-based therapies (Romero-Calle et al., 2019). The development of engineered phages and synthetic endolysins has also reduced the potential for immune responses while broadening their host range. These innovations address challenges such as narrow host specificity, immune system neutralization of phages, and rising bacterial resistance (Sithu Shein et al., 2024).

Phage-antibiotic combination therapy presents a promising approach to enhance efficacy and prevent resistance. In infections caused by *Burkholderia cepacia*, the combination of phages KS12 or KS14 with meropenem improved survival rates in Galleria mellonella models and inhibited resistance development. For *P. aeruginosa*, adjunctive therapy with tobramycin or ceftriaxone enhanced phage penetration into biofilms, leading to significant reduction in bacterial load. In *S. aureus*, the combination of phages and gentamicin increased bactericidal activity by facilitating bacterial aggregation and lysis (Khosravi et al., 2024). In *S. pneumoniae* infections, the combination of phage lysins and antibiotics markedly enhanced survival rates in murine models compared to monotherapies (Fernández et al., 2021; Khosravi et al., 2024). These synergistic effects are particularly pertinent in managing biofilm-associated infections, where conventional antibiotics often prove inadequate. A summary of key pathogens, associated phages, delivery methods, study models and outcomes is provided in Table 3.

**Table 3:** Summary of experimental and clinical evidence supporting phage therapy for respiratory infections.

Pathogen	Phage(s)	Delivery method	Model/Setting	Outcome	Reference
*K. pneumoniae*	Phage 1513	Intranasal (mouse)	Animal model	80% survival at high dose; ↓ cytokines (TNF-α, IL-6), ↓ bacterial burden	Liang et al. (2023)
*P. aeruginosa*	PAK-P1, PAK-P3, phage cocktail	Aerosol, IV	Animal models, a CF patient	Reduced bacterial load; improved lung function	Drulis-Kawa et al. (2015) and Samaee et al. (2023)
*Acinetobacter baumannii*	Bϕ-C62, Ab_SZ3	Aerosol, IV	VAP, COVID-19, COPD patients	Infection eradicated in 2 weeks; recovery in COPD patients	Fernández et al. (2021) and Khosravi et al. (2024)
*S. aureus*	AB-SA01, Pyophage	Inhalation, IV	MRSA pneumonia models, CF case	↓ lung burden; improved survival; no adverse effects reported	Cao et al. (2015), Fernández et al. (2021) and Sithu Shein et al. (2024)
*Mycobacterium abscessus*	Engineered phage cocktail + rifabutin	IV	Post-lung transplant, zebrafish	Cleared infection; improved outcomes with rifabutin	Sithu Shein et al. (2024)
*Burkholderia cepacia*	KS12/KS14 + Meropenem	IV, aerosol	Galleria mellonella (larvae)	Enhanced survival; reduced resistance development	Cao et al. (2015)
*S. pneumoniae*	Cpl-1, Cpl-7, Cpl-711 + antibiotics	IV, intranasal	Mouse model	Enhanced bactericidal effects; synergy with amoxicillin and cefotaxime	Cao et al. (2015) and Fernández et al. (2021)

Preclinical studies have demonstrated promising antibacterial effects of phage therapy against key lung pathogens in animal and *in vitro* models. However, human trials remain limited, and efficacy in clinical practice is still under investigation.

#### Metals

Metals are crucial for bacterial growth and homeostasis, but high concentrations can be lethal (Hood and Skaar, 2012). Metal compounds exhibit stronger antimicrobial effects than organic ones and could enhance antibiotic activity without fostering resistance (Frei et al., 2020; Laxminarayan et al., 2013). This strategy is not unprecedented, as historical applications reveal the use of silver as a wound antiseptic in the 18th and 19th centuries (Alexander, 2009). Key metals possess antimicrobial properties include (Mittapally et al., 2018) silver, zinc, gold, manganese, and copper, which are the focus of this review, detailing their mechanisms against bacteria (Table 4).

**Table 4:** Mechanisms of antimicrobial action of metals.

Metal	DNA damage	Enzyme disruption	ROS generation	Membrane disruption	Energy metabolism interference	Iron metabolism disruption
Silver (Ag)	✓	✓	✓	✓	–	–
Gold (Au)	–		(✓)^*^	✓	–	–
Copper (Cu)	✓	✓	✓	✓	✓	–
Manganese (Mn)	–	–	✓	✓	✓	–
Zinc (Zn)	✓	✓	✓	✓	–	–
Galliun (Ga)	–	(✓)^**^	(✓)^**^	–	(✓)^**^	✓

* Light dependent effect.

** Secondary effect of iron metabolism disruption.

### Hard metals: metals that release ions that disrupt bacterial cells

#### Silver

Silver exhibits multiple mechanisms that inhibit bacterial growth. This occurs through the release of silver ions, which bind to nucleic acids and hinder replication, leading to genetic modification (Barras et al., 2018). Additionally, silver disrupts metabolic pathways by displacing essential metals due to its high binding affinity to sulfhydryl groups, which are part of crystalline residual (Morones-Ramirez et al., 2013; Lansdown, 2006; Arakawa et al., 2001). Furthermore, silver generates reactive oxygen species (ROS), contributing to its bactericidal effects against both Gram-negative and Gram-positive bacteria (Domínguez et al., 2020). However, some bacteria, like *S. aureus*, have developed resistance (Feng et al., 2000).

*In vitro* studies show that silver nanoparticles enhance vancomycin’s effectiveness against *MRSA* and *vancomycin-resistant S. aureus (VRSA)* (Jiang et al., 2021; Awad et al., 2021; Elsawy et al., 2021). Awad et al. (Mohamed, 2019) found that treating infected rat lungs with silver nanoparticles with vancomycin led to nearly complete healing compared to those receiving vancomycin alone, improving effectiveness from 70 to 90% (Mohamed, 2019).

Additionally, a recent study showed that combining colistin with silver nanoparticles yielded greater effectiveness than using colistin alone for treating MDR bacteria causing pneumonia (Yassin et al., 2022). This aligns with previous research showing that silver nanoparticles possess antibacterial properties against *carbapenem-resistant Klebsiella pneumoniae (CRKP)* and MDR strains of *K. pneumoniae* by disrupting cell membranes and inducing a time-dependent accumulation of ROS (Yang et al., 2021; Hassan et al., 2024). Additionally, silver nanoparticles show promise for treating lower respiratory infections, exhibiting activity against carbapenem-resistant *Acinetobacter baumannii (CRAB), CRKP, P. aeruginosa, H. influenzae, MRSA, and S. pneumoniae* (Huang et al., 2022).

Currently, there are no registered Phase I or II clinical trials on clinicaltrials.gov that investigate the use of silver nanoparticles with antibiotics. In vitro studies present a promising approach to combat *MRSA* and other MDR bacterial infections. The only FDA approved silver drug is silver sulfadiazine, a topical antibiotic for burn wounds, due to systemic toxicity (Weir, 1979; Aziz et al., 2012). However, silver has demonstrated its effectiveness in preventing infections when applied to medical devices such as implants, heart valves, urinary and peritoneal catheters, and surgical sutures (Blaker et al., 2004; Song et al., 2009; Vasilev et al., 2009). For example, a silver nanoparticles-coated endotracheal tube can prevent the adhesion of *P. aeruginosa* in a lung model (Hartmann et al., 1999).

#### Gold

The antimicrobial action of gold remains unclear; but, research suggests that it may induce bacterial cells apoptosis through membrane distortion and inhibiting efflux pumps (Rocha et al., 2007; Comtois et al., 2003; Scharf et al., 1998). Gold nanoparticles can generate ROS when exposed to hot electrons or radiation, damaging DNA (Table 3; Nguyen et al., 2025). This suggests their potential as biocompatible antibiotic alternatives for treating infections like pneumonia.

Research indicates gold nanoparticles possess antibacterial properties, giving a promise in combination therapies. In vitro study by Abdelshakour et al. (2023) demonstrated that the combination of gold nanoparticles and vancomycin exhibited a synergistic effect against MRSA (Abdelshakour et al., 2023), and other pathogenes like *E. coli, Klebsiella oxytoca*, and *P. aeruginosa*, even at low vancomycin doses (Hagbani et al., 2022). The use of gold nanoparticles significantly enhances the bactericidal activity of cefixime, increasing it by eightfold, and cuts the time required to combat *S. aureus* in half (Ali et al., 2020). The clinical potential is further supported by FDA Auranofin; an antirheumatic drug derived from Au(I)-phosphine, which has shown antibacterial effects against *S. aureus*, *and MRSA* (Harbut et al., 2015). These findings suggest that gold nanoparticles have significant potential for treating antibiotic-resistant infections, including pneumonia.

#### Copper

Copper protein, DNA, and cell membranes (Su et al., 2015) by generating ROS, which inhibits anaerobic respiration and lead to energy loss and damage DNA and proteins (Table 4; Su et al., 2015; Wang et al., 2019). Recent research show the ability of copper nanoparticles to fight against respiratory bacteria including pneumonia bacterial. Copper oxide nanoparticles significantly reduced *K. pneumonia* growth, suggesting their potential to enhance bacterial pneumonia treatment (Ermini and Voliani, 2021).

The antimicrobial applications of copper extend to combination therapies. For example, the combination of copper-dependent antibacterial compounds and ampicillin can reverse *MRSA* resistance, even at lower concentrations of ampicillin. Interestingly, another study discovered that the presence of copper ions alongside β-lactam antibiotics, such as meropenem, amoxicillin, ampicillin, and ceftriaxone, reduced their antibacterial effectiveness against *E. coli* and *S. aureus*, although this interaction did not occur with Penicillin G (Božić et al., 2018). Copper cluster represents a novel compatible therapy with broad spectrum of antibacterial activity, particularly against MDR Gram-positive and Gram-negative bacteria. Studies demonstrated their effectiveness against *MRSA, S. aureus, E. coli and P. aeruginosa* attributed to their effect on cell wall and ROS production.

#### Gallium

Gallium possesses a distinctive mechanism of action compared to other metals. It targets bacteria by mimicking iron in both its ionic charge and electronic configuration. Because bacteria rely heavily on iron for growth and metabolism, the substitution of gallium for iron interferes with essential iron-dependent processes, leading to functional iron starvation and disruption of critical bacterial activities (Chitambar, 2016).

Xia et al. (2021) demonstrated that a combination of gallium protoporphyrin and gallium nitrate was highly effective against MRSA biofilms. This combination promoted biofilm disintegration through an extracellular DNA-dependent mechanism, converting mature, antibiotic-tolerant biofilms into immature forms that are more susceptible to treatment. When followed by vancomycin, these altered biofilms were eradicated within 1 week using low antibiotic concentrations (Xia et al., 2021). Further evidence from a recent study on gallium nanoparticles confirmed these findings. Gallium-doped bioactive carbon dots exhibited stronger antibacterial activity against *E. coli* and superior biofilm disruption compared with undoped carbon dots, while maintaining high compatibility with human cells (Radha et al., 2025). A phase I clinical trial evaluating intravenous gallium in patients with cystic fibrosis and chronic *P. aeruginosa* infection reported improved lung function with no adverse effects and no significant changes in blood cell counts, electrolytes, or kidney function (Goss et al., 2018).

In summary, with gallium having advanced to phase I clinical trials with encouraging results, it represents a promising therapeutic metal with potential applications in the treatment of bacterial pneumonia and other infections caused by MDR pathogens.

#### Essential metals: metals that are important for bacteria in trace amounts but could be toxic in high amounts

#### Manganese

Despite being toxic at high concentrations, manganese is thought to be a crucial ion for bacteria (McCarron et al., 2018). Manganese exhibits a multi-targeted antimicrobial effect by disrupting redox balance and contributing to ROS accumulation, interfering with ATPase function, and compromising membrane integrity, ultimately leading to bacterial cell death (Du et al., 2020).

The therapeutic potential of manganese-based formulations has been demonstrated by recent studies. Silver-doped manganese dioxide nanoparticles showed their effectiveness against *MRSA*, lead-resistant *P. aeruginosa*, *Streptococcus epidermis*, *E. coli*, and *K. pneumoniae* by disrupting resistance and biofilm in lungs (Kunkalekar et al., 2013; Kunkalekar et al., 2014).

A promising 2023 study examined the effectiveness of manganese nanoparticles vaccine for bacterial pneumonia. The results are promising as the vaccine robust immune response through the production of antigen-specific IgG antibodies, providing complete protection for mice against *Yersinia pestis* infection (OuYang et al., 2023).

To this point, there has been a lack of research examining the role of manganese in conjunction with antibiotics to increase their efficacy or to mitigate the resistance of multidrug bacteria.

#### Zinc

Zinc is essential for bacterial cell function, but excessive absorption can result in cytotoxicity (Borovanský and Riley, 1989). It combats bacteria by generating ROS, disrupting cell membranes, and interfering with enzymes and DNA replication by releasing Zn^2+^ ions in aqueous environments (Table 4; Borovanský and Riley, 1989; Fang et al., 2010; Li et al., 2011).

Limited research on using zinc for pneumonia pathogens. However, in murine models treated with zinc nanoparticles for bacterial pneumonia, a significant reduction in *CRKP* infection was observed due to the generation of ROS and broad-spectrum antibacterial properties (Liu et al., 2022). In another study, zinc nanoparticles inhibited the growth of MDR *Acinetobacter baumannii* and affected its biofilm formation that were isolated from pneumonia patients (Hou et al., 2020).

The findings highlight the potential of zinc-based treatments for bacterial infections and combating antibiotic resistance. Zinc nanoparticles offer a multifaceted approach by directly targeting bacteria and aiding host healing. Combining zinc with traditional antibiotics may improve treatment outcomes, especially for resistant strains. Future research should focus on optimal formulations and delivery methods to maximize zinc’s benefits while reducing cytotoxicity, aiming to enhance treatments for bacterial infections and advance public health.

Drawing from recent research in the reviewed literature, metals like silver, copper, and zinc have significant antimicrobial properties that may help reduce AMR, especially against pneumonia-causing bacteria. However, their toxicity to humans and potential for inducing resistance require careful management. Metal nanoparticles combined with antibiotics show promise in combating MDR bacteria, but most evidence comes from *in vitro* or animal studies, necessitating clinical trials for validation. Strategies include developing stimulus-responsive coatings to minimize toxicity and reviving inactive antibiotics with metal to restore efficacy. Long-term studies on metal nanoparticle accumulation in wastewater are also needed. Addressing these issues could position metal-based solutions as vital in the fight against AMR.

#### Probiotics

Probiotics may treat or prevent infections and inflammation by enhancing immune responses and mucosal barrier function (Najafi et al., 2021). They work by inhibiting pathogens, blocking bacterial translocation, enhancing immune responses via toll-like receptor pathways, and improving gut mucosal barrier integrity (Cheema et al., 2022; Han et al., 2024). Probiotics also produce antimicrobial substances, such as short-chain fatty acids and bacteriocins, which aid in their therapeutic effects.

Mechanistically, probiotics exert bidirectional immune regulation; they stimulate host defense by enhancing macrophage and natural killer cell activity, while simultaneously suppressing excessive inflammation by downregulating pro-inflammatory cytokines (e.g., IL-6, TNF-α) and upregulating anti-inflammatory mediators like IL-10. This balanced modulation helps restore mucosal immune homeostasis during infection (Najafi et al., 2021; Liu et al., 2018).

Recent studies indicate they could manage pneumonia, particularly VAP, by balancing microorganisms in the aerodigestive tract, modulating immune response, and lowering inflammatory cytokines like IL-6 and TNF-α (Sun et al., 2022). A RCT (Najafi et al., 2021) involving 100 hospitalized bacterial pneumonia patients assessed the effectiveness of probiotics. Participants were divided into probiotic and placebo groups, primarily treated for CAP. The probiotic group received two sachets daily for 5 days, containing *Lactobacillus rhamnosus, Lactobacillus casei, Lactobacillus acidophilus (L. acidophilus), Lactobacillus bulgaricus, Bifidobacterium breve, B. infantis, Streptococcus thermophilus*, and the prebiotic fructo-oligosaccharide. Both groups received the same antibiotics; ceftriaxone and azithromycin. Results revealed that administering probiotics significantly reduced the duration of critical symptoms (dyspnea, tachypnea, cough, fever, and crackles) and hospitalization length. These results suggest that probiotics could enhance treatment outcomes and reduce the risk of complications associated with pneumonia when used with antibiotics. These findings suggest that probiotics can enhance recovery by modulating inflammation rather than directly eradicating pathogens.

Another RCT study, Adinatha et al. (2020), involved 54 children aged 2 months to 5 years with severe CAP. All received standard intravenous ampicillin and gentamicin; with half also receiving a five-day probiotic *mix*, while the other half had a placebo. Baseline characteristics were similar across groups. Results showed no significant differences in hospital stay, or fever duration. However, the probiotic group had shorter rales (72 h vs. 84 h; *p* = 0.037), and multivariate analysis indicated probiotics reduced rales by 5.87 h (*p* = 0.022). Overall, while probiotics did not shorten hospital stays or reduce fever, they may help resolve pulmonary crackles without safety concerns. These results point to an immune-stabilizing role rather than an antimicrobial one, helping accelerate lung recovery through inflammation control.

Furthermore, a RCT assessed probiotics effectiveness in treating 80 young hospitalized adults with mild CAP with standard antibiotics (ceftriaxone, azithromycin, and then fluoroquinolones) with or without probiotics (*Bacillus subtilis* and *Enterococcus faecium*). Results showed that the probiotic group experienced shorter fever duration (2.3 ± 0.7 days vs. 3.3 ± 1.3 days; *p* = 0.003), improved general health (VAS score: 7.8 vs. 6.9; *p* = 0.007), better stool quality (abnormal stool rate: 4.2% vs. 37.9%; *p* = 0.009), and 100% normalization of hs-CRP levels by week 2 versus 74.1% (*p* = 0.023). Immunological analysis indicated higher regulatory T cells (Tregs) and lower Th17/Treg levels in the probiotic group, with no serious side effects were reported. Overall, probiotics may alleviate CAP symptoms, modulate the immune system, and speed up inflammation resolution, although, the study was limited to young military personnel with mild disease. This supports the immunoregulatory hypothesis that probiotics promote anti-inflammatory balance via Treg activation and cytokine modulation.

A systematic review and meta-analysis (Han et al., 2024) of 13 studies (2010–2023) including 795-5,543 critically ill patients on mechanical ventilation for at least 48 h who were at risk for VAP. Eleven of these studies indicated that probiotics significantly reduced VAP incidence compared to placebo or standard care, and were associated with shorter ICU stays and reduced mechanical ventilation time. However, caution is advised, and further high-quality, standardized research is needed to validate these findings. Another meta-analysis (Zhao et al., 2021) of 15 RCTs involving 2039 ICU patients on mechanical ventilation assessed the impact of probiotics on preventing VAP. Results showed a significantly lower risk of VAP in the probiotic group compared to placebo. However, probiotics did not affect mechanical ventilation duration, ICU stay, or mortality rates at 28 days or 90 days. Variability in study quality was noted, with subgroup analysis indicated that probiotics were more effective in patients meeting non-quantitative microbiological criteria (RR = 0.63; 95% CI: 0.47–0.83; *p* = 0.001). While probiotics may reduce VAP risk, they do not influence severe clinical outcomes, emphasizing the need for large, multicenter trials. Despite heterogeneity in strains, dosing, and protocols, the collective evidence supports probiotics as adjunctive immunomodulators rather than stand-alone antimicrobials.

The literature indicates that probiotics may help manage respiratory infections, particularly in critical care settings by reducing symptoms and preventing complications like VAP. Their use requires careful consideration of benefit against research limitations, like strains differences, and dosages variability. Probiotics are generally safe and can complement conventional pneumonia therapies, and improve patient outcomes while potentially reducing antibiotics reliance. However, more rigorous, large-scale studies are needed to establish guidelines for their optimal clinical use.

#### Vaccines

Vaccines are vital in combating AMR by preventing infections, thereby reducing antibiotic use and the prevalence of resistant bacteria (Jansen et al., 2021). They enhance herd immunity, particularly in preventing bacterial pneumonia among vulnerable populations like elderly and children (Micoli et al., 2021; Daraghmeh et al., 2025). Vaccines are highly specific and less likely to induce resistance compared to antibiotics, making them suitable for large-scale preventive measures. The 23-valent polysaccharide vaccine (PPV23) and 13-valent conjugate (PCV13) vaccine have been shown effectively to reduce pneumococcal disease rates (Berild et al., 2020). Conjugate vaccines, in particular, have decreased disease incidence and hospitalizations through herd immunity, although they may contribute to the emergence of resistant serotypes. While vaccination has proven effective in developed countries, challenges such as low awareness and high costs hinder adult vaccination (Micoli et al., 2021; Daraghmeh et al., 2025).

A systematic review found that 23 of 26 studies reported significant reductions in antibiotic use following influenza or pneumococcal vaccination (Doherty et al., 2020), highlighting the importance of vaccination programs in combating AMR and antimicrobial stewardship.

Vaccines against bacterial pathogens can protect against antibiotic-resistant infections, but may also influence antibiotic resistance dynamics among unvaccinated individuals. Understanding this requires knowledge of the factors driving selection for resistant strains. Using mathematical modeling and data from 2007 on penicillin use and resistance in *S. pneumoniae* across 27 European countries, Davies et al. (2021) identified factors like antibiotic use, diversity and strain competition that explain penicillin resistance frequency. The models predicted that non-serotype-specific pneumococcal vaccination impact on disease incidence, and antibiotic resistance depends significantly on the competition between drug-resistant and sensitive strains. Additionally, country-specific transmission differences, indicating that resistance management policies must be tailored to specific pathogens and contexts.

Similarly, Tithi et al. (2023) analyzed drug-susceptible and drug-resistant pathogens during a double-dose vaccination campaign, identifying two basic reproduction numbers: R01 for drug-susceptible strains and R02 for drug-resistant strains. The study found that if max [R01, R02] > 1, a strain can spread; if both are <1, the disease fades. Four equilibrium points were identified, and stability was assessed using the Routh-Hurwitz criteria. Notably, poor vaccine quality can increase drug-resistant strain prevalence despite timely vaccination of drug-susceptible strains. Sensitivity analysis showed that transmission rates significantly impact disease outbreaks, and effective vaccination programs can reduce the burden of both strains (Tithi et al., 2023). These studies emphasize the need to consider strain competition in vaccination strategy design to manage antibiotic resistance.

For effective vaccination programs, policymakers should prioritize accessibility and affordability, particularly in resource-limited areas, to ensure widespread immunization. Public awareness campaigns are important for educating communities about vaccine benefits in preventing diseases and reducing antibiotic resistance. Ongoing research and innovation in vaccine development, combined with collaborative efforts among governments, healthcare providers, and the pharmaceutical industry are vital for addressing infectious diseases and antimicrobial challenges. Integrating vaccination into antimicrobial stewardship provides direct health benefits and helps combat AMR, protecting public health for future generations.

#### Natural remedies

In the search for new therapies against AMR, traditional and herbal medicines have garnered increasing scientific interest for their antibacterial potential. In what is now referred to as the “post-antibiotic era,” these natural remedies are being explored for treating infections like bacterial pneumonia (Abdallah et al., 2023; Patel and Sharma, 2021; Duțu et al., 2022).

Over the last decade, research into herbal remedies has shifted from simply documenting traditional use to scientifically investigating their mechanisms of action. For a complex illness like bacterial pneumonia, the focus is rarely on finding a single herb to replace antibiotics. Instead, recent advances concentrate on a few key areas:

- Adjunctive Therapy: combining herbs with antibiotics improves clinical outcomes, as shown by meta-analyses on Chinese Herbal Medicine (CHM), which is more effective against drug-resistant pneumonia (Zhao et al., 2023; Guo et al., 2022).
- Immunomodulation: Modifying the body’s immune response to help it fight infection more effectively without causing excessive, damaging inflammation (e.g., a “cytokine storm”). Advanced approaches like “material-herbology” have shown that plant-derived nanodots can calm cytokine storms, representing a cutting-edge form of immunomodulation (Li et al., 2021).
- Targeting AMR: Investigating plant compounds that weaken bacteria by disrupting their defence mechanisms, making them more susceptible to conventional antibiotics. These mechanisms include disrupting the bacterial membrane, inhibiting essential enzymes, and blocking quorum sensing and biofilm formation (Abdallah et al., 2023).
- Symptomatic Relief: Using herbs to manage symptoms like cough, chest congestion, and fever, thereby improving patient comfort and accelerating recovery. Clinical studies have validated this by showing that CHM can reduce cough duration and lung rales in children with pneumonia (Guo et al., 2022).

This ethnobotanical knowledge, spanning diverse geographical regions, serves as a rich foundation for modern scientific investigation into new treatments for respiratory infections.

Systematic reviews of traditional medicine have documented a wealth of plant species used across the globe for treating pneumonia and related respiratory symptoms. This ethnobotanical evidence is the crucial first step in identifying candidates for pharmacological validation.

- In Kenya, a systematic review identified 105 medicinal plants from 43 families with documented in-vitro activity against human pathogenic bacteria. Plants from the Lamiaceae, Rutaceae, and Fabaceae families were most common, with species such as *Toddalia asiatica*, *Hagenia abyssinica*, and *Warbugia ugandensis* demonstrating the strongest antimicrobial activities (Odongo et al., 2022).
- In the Himalayan Region, a comprehensive review documented 137 plants traditionally used for pneumonia and tuberculosis. The most frequently cited plant families were Asteraceae, Bignoniaceae, and Fabaceae. Of these, *Curcuma longa* L. (turmeric), *Punica granatum* L. (pomegranate), and *Justicia adhatoda* L. showed the most potent inhibitory activity against *S. aureus* and *S. pneumoniae* (Patel and Sharma, 2021; Adnan et al., 2019).
- In Assam, India, a study validating local folk medicine found that 14 out of 20 traditionally used plants showed antibacterial activity against pneumonia pathogens. Notably, extracts of *Mucuna pruriens* and *Xanthium strumanium* exhibited stronger antibacterial activity against *S. pneumoniae* than the control antibiotic, chloramphenicol, providing direct scientific evidence for their traditional use (Kalita and Kalita, 2014).
- In Pakistan, an experimental study focused on plants from the Jahangira area, such as *Azadirachta indica* (Neem), *Vachellia nilotica*, and *Allium sativum* (Garlic), demonstrating their strong antibacterial properties against clinical isolates of *S. pneumoniae* (Bibi and Waqas, 2024).

Beyond ethnobotany, a growing body of *in-vitro*, *in-vivo*, and clinical research has begun to validate the efficacy of these traditional remedies. Meta-analyses of CHM, in particular, have provided high-level evidence for their role in managing bacterial pneumonia.

A meta-analysis of 38 RCTs involving 2,890 patients with MDR or XDR bacterial pneumonia found that CHM combined with antibiotics was significantly superior to antibiotics alone. The combined therapy improved the clinical response rate and microbiological eradication, reduced inflammatory markers (WBC, procalcitonin, CRP), and shortened the length of hospitalization (Zhao et al., 2023). A separate meta-analysis on pediatric pneumonia convalescence showed that CHM also accelerated recovery by reducing cough relief time and the time for lung rales to disappear (Guo et al., 2022).

A key advancement is the shift from studying crude extracts to identifying the specific bioactive compounds (phytochemicals) responsible for their therapeutic effects. Various studies have isolated compounds with potent antibacterial properties relevant to pneumonia:

- Key Phytochemicals: Across multiple reviews, compounds like curcumin (from *Curcuma longa*), vasicine (from *Justicia adhatoda*), piperine, quercetin, myricetin, and gallic acid have been repeatedly identified as having strong antibacterial properties against pneumonia-causing pathogens (Patel and Sharma, 2021; Adnan et al., 2019).
- Multifaceted Mechanisms: Unlike conventional antibiotics that often have a single target, phytochemicals can combat bacteria through multiple mechanisms, making it harder for resistance to develop. These include disrupting the bacterial membrane, inhibiting essential enzymes, interfering with protein synthesis, and blocking quorum sensing and biofilm formation (Abdallah et al., 2023).

Recent research has moved into highly innovative areas, merging traditional medicine with cutting-edge technology to create novel and highly effective therapies.

- Network Pharmacology: This computational biology approach is being used to deconstruct the complex, multi-component, and multi-target nature of traditional herbal formulas. Studies using network pharmacology have predicted the key targets and signaling pathways (e.g., MAPK, TNF, PI3K-Akt) through which Chinese patent medicines like Xuebijing injection and Lianhuaqingwen capsules treat pneumonia. This allows for a mechanistic understanding that aligns with modern biology while respecting the holistic principles of traditional medicine (Yang et al., 2025; Wu and Zhong, 2021).
- Material-Herbology: A groundbreaking strategy has emerged that treats herbal medicines as functional biomaterials. In a pioneering study by Li et al. (2021), researchers fabricated “tea nanodots” (TNDs) of ~3 nm from black tea catechins. These TNDs demonstrated a multi-pronged ability to eradicate lethal viral-bacterial pneumonia (H1N1-MRSA) in animal models. Their mechanisms included; Antibacterial Action, which involves physically disrupting the *MRSA* membrane and inhibiting its amino acid metabolism. Secondly, antiviral Action, involving blocking the active site of the H1N1 neuraminidase enzyme and anti-inflammatory action which entails calming the associated cytokine storm. This approach, which combines the therapeutic properties of herbs with the precision of materials science, resulted in a 100% recovery rate from a lethal co-infection, showcasing a highly effective and safe future direction for pneumonia treatment (Li et al., 2021).

A synthesis of the literature revealed several key limitations in the current body of research and points toward clear recommendations for future work.

- The Ethnobotanical to Scientific Gap: While there is a vast repository of ethnobotanical knowledge on plants used for pneumonia and other respiratory illnesses, only a small fraction has been scientifically validated. Previous reviews highlight a major research gap between traditional use and pharmacological validation (Odongo et al., 2022; Adnan et al., 2019).
- Lack of *In-Vivo* and Toxicological Data: A significant limitation repeatedly mentioned is that most research is confined to *in-vitro* studies. There is a marked scarcity of *in-vivo* animal studies and almost no data on the toxicology, pharmacokinetics, and mechanisms of action for the vast majority of traditionally used plants. This is a critical barrier to translating promising lab results into safe and effective therapies (Adnan et al., 2019; Mishra et al., 2024).
- Need for High-Quality Clinical Trials: Even where clinical research exists, particularly for Chinese Herbal Medicine, meta-analyses point out limitations in the primary studies, such as small sample sizes, inadequate blinding, and lack of protocol registration. The consistent recommendation is for larger, multi-center, well-designed, and rigorously reported RCTs to provide more robust clinical evidence (Zhao et al., 2023; Guo et al., 2022).
- Future Directions involve combining traditional knowledge with modern Science: Recommendations include leveraging advanced “omics” technologies (genomics, proteomics, metabolomics) and computational approaches like network pharmacology to deconstruct the complex, multi-target nature of herbal formulas. This will help identify active compounds and understand their precise mechanisms, moving beyond simple extract screening (Abdallah et al., 2023; Yang et al., 2025).

The status of natural and herbal remedies for bacterial pneumonia has progressed from an ethnobotanical knowledge scientific and clinical validation. While crude extracts from diverse global flora show promise, the forefront of research lies in understanding the complex, multi-target mechanisms of herbal formulas and leveraging modern technology. Overcoming the current limitations requires a concerted effort to bridge the gap between traditional use and scientific evidence through more robust *in-vivo*, toxicological, and high-quality clinical studies. Advanced approaches like network pharmacology and material-herbology are providing unprecedented insights and creating novel therapeutic strategies. These advancements signal a paradigm shift, positioning natural products not merely as alternatives, but as a sophisticated and highly effective source for the next generation of therapies to combat the growing threat of drug-resistant bacterial pneumonia.

Most studies on herbal extracts and natural compounds remain in the experimental phase, with limited translational data. Their antimicrobial and immunomodulatory prospects are promising, but validated clinical efficacy has yet to be demonstrated in human trials.

## Limitation of proposed alternatives

Despite promising preclinical and early clinical results, several limitations remain for these alternative therapies. Stem cell–based approaches face delivery challenges and risks such as immune rejection, fibrosis, and ectopic differentiation. Bacteriophage therapy is constrained by narrow host specificity and potential immune neutralization. Metal-based nanoparticles may raise concerns regarding cytotoxicity and bioaccumulation, while probiotics suffer from strain variability and inconsistent dosing. Herbal therapies face standardization and reproducibility challenges, whereas gene-editing strategies such as CRISPR–Cas systems require further evaluation of off-target and biosafety effects. Addressing these limitations through standardized protocols and long-term clinical evaluation is crucial for their safe clinical translation.

## Discussion

The treatment landscape for bacterial pneumonia is undergoing a pivotal transformation as the limitations of conventional antibiotics become increasingly evident. This review highlights the breadth and depth of emerging alternatives- from biological (stem cells and bacteriophages, probiotics, vaccines) to material based (metals, nanoparticles), to genetic and molecular (CRISPR-Cas systems) and herbal medicines—that offer innovative and multifaceted strategies to combat antimicrobial resistance. These approaches do not merely serve as replacements but bring new mechanisms, enhance host immunity, and hold potential for synergistic use alongside antibiotics. While many of these strategies are still in early stages of validation, their collective promise marks a significant step toward reshaping pneumonia management. Continued interdisciplinary research, clinical trials, and global cooperation will be critical to translating these advancements into effective, accessible, and sustainable therapies for the future.

The strength of this review lies in its comprehensive integration of diverse therapeutic strategies against AMR in bacterial pneumonia, offering a multidimensional understanding that bridges biological, material, and molecular innovations. However, one limitation is the heterogeneity of available evidence, as many studies remain at preclinical or early clinical stages with limited standardized data, which may affect generalizability. Future research should emphasize rigorous clinical validation and long-term safety evaluation of these alternatives, particularly regarding delivery mechanisms and host–microbe interactions.

Moreover, AI offers an emerging opportunity in AMR research and drug discovery. AI-assisted modeling, including *de novo* AMP design, holds potential to optimize peptide structure, enhance antibacterial selectivity, and overcome limitations of natural AMPs through rational design. Such integration of computational and experimental approaches may accelerate the discovery of next-generation antimicrobials and complement traditional laboratory-based efforts.

Different alternative therapies are likely to have different roles depending on the clinical setting. Rapid-acting interventions such as bacteriophages, nanoparticles, and metal-based antimicrobials may be most useful for acute and severe infections, while longer-term or preventive options—like probiotics, herbal formulations, or stem cell therapies—may be better suited for chronic inflammation control and prevention. Future work should focus on identifying how these approaches can complement each other, be standardized for clinical use, and address resistance mechanisms more effectively.
