# Supplementary material for: Advancements in alternative approaches to address antimicrobial resistance in bacterial pneumonia: a comprehensive review
Source: Front Microbiol. 2025 Nov 19;16:1704931. doi: 10.3389/fmicb.2025.1704931 (PMC12673510; doi:10.3389/fmicb.2025.1704931)
Supplement: Supplementary file 1 [file Table_1.docx]

Advancements in alternative approaches to address antimicrobial resistance in bacterial pneumonia: A Comprehensive Review

# Supplementary tables:

**Supplemmantary table 2** Antibiotic classes, uses and resistance mechanisms in pneumonia treatment.

| **Class** | **Subclass, example** | **Common use** | **Resistance mechanism** | **Gene** |
| --- | --- | --- | --- | --- |
| **Beta-lactams** | Penicillins, Cephalosporins, Carbapenems, Monobactams, Ceftobiprole *(Novel)*, Ceftaroline *(Novel),* Ceftolozane/tazobactam *(Novel)* | First-line for CAP and HAP; broad-spectrum coverage | Altered PBP, β-lactamases production | mecA, mecC, mecB, mecD (encode altered P bacterial pneumonia s), bla genes (encode β-lactamases. |
| **Macrolides** | Azithromycin, Clarithromycin,  Erythromycin,  Solithromycin *(Novel)* | Targeting atypical pathogens, often combined with beta-lactams | rRNA methylation, Efflux pumps | erm genes (encode methylation), mef(A/E) ( encodes efflux pumps) |
| **Fluroquinolones** | Levofloxacin,  Moxifloxacin,  Gemifloxacin,  Delafloxacin *(Novel)* | Used as monotherapy in CAP or resistant cases; covers atypicals | Mutations in DNA gyrase (GyrA, GyrB) and topoisomerase IV, Efflux pumps | gyrA, gyrB, parC, ParE and qnr genes |
| **Tetracyclines** | Doxycycline, Minocycline | Used in mild CAP; good for atypical organisms | Efflux pumps,  Ribosomal protection proteins, and Enzymatic inactivation | tet(A/B/K/L/M/O) |
| **Aminoglycosides** | Gentamicin, Amikacin, Tobramycin | Severe HAP; often in gram-negative infections | Methylation of rRNA, and AME | aac, aph, ant (AMEs), armA, rmtA–E (rRNA methyltransferases) |
| **Glycopeptides** | Vancomycin, Teicoplanin | Treatment of MRSA pneumonia | Alteration of peptidoglycan precursors | vanA, vanB, vanC, vanD |
| **Oxazolidinones** | Linezolid | Alternative to vancomycin for MRSA and VRE | Methylation of 23S rRNA,  Mutations in ribosomal proteins | cfr (23S rRNA methyltransferase), optrA, poxtA (ribosomal protection) |
| **Polymyxins** | Colistin (Polymyxin E), Polymyxin B | Last-resort for MDR gram-negative | Modification of lipid A in LPS | mcr genes |
| **Pleuromutilin** | Lefamulin *( Novel; first-in-class)* | CAP caused by typical bacteria; a alternative to macrolides | - | - |

PBP: penicillin-binding proteins, AME: Aminoglycoside-modifying enzymes
